# mLearning- a cost effective initiative for stigma reduction in health care settings

**DOI:** 10.1186/1471-2334-14-S3-E46

**Published:** 2014-05-27

**Authors:** Sree T Sucharitha, Janani Rajagopal, Ravi Siriki

**Affiliations:** 1 Department of Community Medicine, Tagore Medical College Hospital, Chennai, India; 2 Independent Data Consultant, Hyderabad, India

## Background

The total number of people living with HIV (PLHIV) in India estimated to be around 20.9 lakhs in 2011. In Indian hospitals, stigma and discrimination is widespread and has unfavorable impact on treatment outcomes for PLHIV and on battle against HIV epidemic. mLearning is a type of mHealth for educating and increasing the capacity of health care providers. The objective of this study was to assess the effect of mLearning intervention as improvement in knowledge and attitudes in medical undergraduates towards reducing stigma in health care settings.

## Methods

This observational cohort study involved mLearning intervention using the free version of WAY2SMS with thirty short messages (SMS) being sent to thirty medical undergraduates of Tagore Medical College, Chennai during August-October 2013.

## Results

Fear of contagion (33%) and poor awareness (23%) among care providers is identified as reasons for stigma. Improved knowledge about definition of stigma as 100%, 97% for Quality, Quantity of HIV virus and Route of transmission (QQR) determining the risk of HIV transmission, 97% for identifying scheduling consultancies till last and charging high for PLHIV surgeries as stigma, was seen in mLearning group. Positive attitudinal shift was seen as 100% participants mentioning they would befriend and would not be ashamed being care providers for PLHIV, 47% realizing the importance of client informed, voluntary testing for HIV, and 60% favored informed consent prior to HIV testing.

## Conclusion

mLearning is a cost effective method of reducing stigma in health care settings by improving knowledge and attitudes of care providers.

